# Interviewing Suspects in Denial: On How Different Evidence Disclosure Modes Affect the Elicitation of New Critical Information

**DOI:** 10.3389/fpsyg.2017.01154

**Published:** 2017-07-17

**Authors:** Lennart May, Pär Anders Granhag, Serra Tekin

**Affiliations:** ^1^Department of Psychology, University of Kiel Kiel, Germany; ^2^Norwegian Police University College Oslo, Norway; ^3^Department of Psychology, University of Gothenburg Gothenburg, Sweden

**Keywords:** suspect interview, information elicitation, information gathering, counter-interrogation strategies, strategic use of evidence

## Abstract

This study examines how different evidence disclosure modes affect the elicitation of new critical information. Two modes derived from the Strategic Use of Evidence (SUE) framework were compared against an early disclosure mode (i.e., the evidence was disclosed at the outset of the interview). Participants (*N* = 88) performed a mock crime consisting of several actions before they were interviewed as suspects. In both SUE conditions the interviewer elicited and disclosed statement-evidence inconsistencies in two phases after an introductory phase. For the SUE-Confrontation (SUE-C) condition, the interview was introduced in a business-like manner, and the interviewer confronted the suspects with the in/consistencies without giving them a chance to comment on these. For the SUE-Introduce-Present-Respond (SUE-IPR) condition, the interviewer introduced the interview in a non-guilt-presumptive way, presented the in/consistencies and allowed the suspects to comment on these, and then responded to their comments; at all times in a non-judgmental manner. Both SUE conditions generated comparatively more statement-evidence inconsistencies. The SUE-IPR condition resulted in more new critical information about the phase of the crime for which the interviewer lacked information, compared to the Early disclosure condition. A likely explanation for this was that (for the SUE-IPR condition) the interviewer used the inconsistencies to create a fostering interview atmosphere and made the suspects overestimate the interviewer's knowledge about the critical phase of the crime. In essence, this study shows that in order to win the game (i.e., obtaining new critical information), the interviewer needs to keep the suspect in the game (i.e., by not being too confrontational and judgmental).

## Introduction

One important aim of a suspect interview is to collect new case-related information (Memon et al., 2003). Ethical interviewing approaches suggest gathering this information in an open minded-manner (e.g., the PEACE model; Bull, 2014). Furthermore, the research literature shows that ethical and humane interviewing approaches are associated with forthcoming suspects (Holmberg and Christianson, 2002; Kebbell et al., 2010; Snook et al., 2015). In suspect interviews, the use of evidence is often at the core. Unfortunately, to date only few studies have provided knowledge on the link between evidence disclosure and information elicitation (e.g., Tekin et al., 2015; Walsh and Bull, 2015). Therefore, this study was designed to examine how different evidence disclosure modes affect the elicitation of new critical information.

### The strategic use of evidence framework

The Strategic Use of Evidence (SUE) framework consists of general principles that can be used to obtain diagnostic cues to deceit (e.g., Hartwig et al., 2014), and to elicit new information (e.g., Tekin et al., 2016). At the core of the SUE framework is the suspects' perceptions of the interviewer's knowledge, and how these perceptions affect the suspects' counter-interrogation strategies, and in turn their verbal responses (Granhag, 2010; Granhag and Hartwig, 2015).

Research has shown that innocent and guilty suspects aiming to convince the interviewer of their innocence differ with respect to their counter-interrogation strategies. Broadly speaking, innocent suspects have seldom something to conceal, they trust that they will be believed if they “just tell it like it happened” (Kassin, 2005), and hence employ forthcoming counter-interrogation strategies (Hartwig et al., 2010). In contrast, guilty suspects are typically motivated to conceal crime-relevant information. Therefore, they commonly prepare for the interview (Hartwig et al., 2007), and reflect on the interviewer's possible knowledge (e.g., Moston and Engelberg, 2011). If they estimate that the interviewer does not hold specific information, they will likely use withholding strategies with respect to this information (“I will not tell any information that might be incriminating”). However, if a guilty suspect believes the interviewer holds specific information, s/he will likely employ forthcoming strategies with respect to this particular information (“It is meaningless to withhold what the interviewer already knows”). Therefore, an interviewer might profit from having the suspect overestimate how much information s/he holds.

An interviewer who aims to elicit new information on a phase of a crime for which s/he lacks information can exploit the knowledge about suspects' counter-interrogation strategies by using the available evidence strategically. For example, the interviewer may elicit statement-evidence inconsistencies by asking questions about the available evidence without disclosing it. Guilty suspects will likely produce statements that are inconsistent with the evidence, as they are unaware of the interviewer's knowledge and therefore tend to use withholding strategies. Next, the interviewer may disclose these inconsistencies to the suspect. This may result in the suspects realizing that the interviewer held more knowledge than first thought. Furthermore, the suspect may rethink his or her perception of the interviewer's knowledge concerning the information that have not yet been discussed (“S/he may hold information also on other aspects”). Basically, the interviewer wants to achieve two things by eliciting and disclosing inconsistencies: (1) to reveal his or her interview tactic to the suspect (i.e., asking questions about the evidence before disclosing it), and (2) to make the suspect overestimate his or her knowledge. Critically, this in turn may result in the suspects changing from less to more forthcoming counter-interrogation strategies. Finally, the interviewer asks questions about the part of the crime for which s/he lacks information. If a guilty suspect now uses more forthcoming counter-interrogation strategies, s/he will reveal new information.

In contrast, innocent suspects are expected to typically use forthcoming counter-interrogation strategies throughout the interview. Therefore, the innocent suspects will likely provide statements that are consistent with the evidence (when asked questions about the evidence) and reveal new information (when asked questions about the part for which the interviewer lacks information).

### Research on the SUE framework

In the first study examining how the SUE framework could be used to elicit new information from guilty suspects, a SUE confrontation condition was compared against an Early disclosure condition (Tekin et al., 2015). To illustrate the implementation of the SUE protocol, consider a crime that can be divided into three different phases (A, B, and C). The interviewer holds evidence about Phase A and B indicating the suspect's possible involvement in the crime. However, the interviewer lacks information about the critical phase (Phase C). The interviewer starts by asking questions about Phase A without disclosing the evidence on this phase (in order to generate statement-evidence inconsistencies), and confronts the suspect with these statement-evidence inconsistencies. The interviewer then repeats this procedure for Phase B. Finally, the interviewer asks about Phase C, for which s/he lacks information. Importantly, in the study by Tekin et al. (2015), for the SUE confrontation condition, the suspects were not given any opportunity to comment on their in/consistencies. For the Early disclosure condition, the interviewer disclosed the evidence about Phase A and Phase B before asking questions about it. The SUE confrontation condition resulted in more statement-evidence inconsistencies, more new information, and that the suspects perceived the interviewer to have held more information about the critical phase of the crime compared to the Early disclosure condition.

In a second study, two SUE conditions were compared against the Early disclosure condition (Tekin et al., 2016). The two SUE protocols were implemented as described above and differed only with respect to the way in which the statement-evidence in/consistencies were handled. For the SUE confrontation condition the suspects were not given any opportunity to comment on the in/consistencies. In contrast, for the SUE confrontation/explain condition, the interviewer explicitly asked the suspects to explain their inconsistencies. Both SUE conditions generated more statement-evidence inconsistencies compared to the Early disclosure condition. However, only the SUE confrontation condition resulted in more new information compared to the Early disclosure condition. This was unexpected as the suspects in both SUE conditions perceived the interviewer to hold comparatively more information about the critical phase. Further analysis showed that a small group of suspects in the SUE confrontation/explain condition were reluctant to explain their inconsistencies, and these suspects revealed less new information compared to the suspects who did explain their inconsistencies. Differently put, some suspects seemed to have given up trying to explain their inconsistencies, and therefore continued to use a withholding strategy also when questioned about the critical phase. This shows that in order to have suspects reveal new information at a later stage in an interview, they have to be willing to explain the inconsistencies that occur early on in the interview.

### The present study

The present study advances research on the SUE framework by examining the effects of three different modes of evidence disclosure. Two modes derived from the SUE framework were compared against a mode for which the evidence was disclosed early in the interview. All interview protocols were divided into an introductory phase (Phase 1) and three questioning phases (Phases 2, 3, and 4). For the *Early disclosure condition*, the interviewer disclosed all the evidence at the outset (Phase 1), and then continued with open-ended invitations asking the suspect to explain the disclosed evidence (Phases 2 and 3). In both SUE conditions the interviewer attempted to elicit statement-evidence in/consistencies for Phases 2 and 3 (by asking questions about the evidence without disclosing it). The SUE conditions differed with respect to the way the interviewer (a) introduced the interview (Phase 1) and (b) disclosed the in/consistencies to the suspects (Phase 2 and 3). For the *SUE-Confrontation* (SUE-C) *condition*, the interviewer introduced the interview in a business-like manner (Phase 1), and confronted the suspects with their in/consistencies without giving them any chance to comment on them (Phases 2 and 3). In contrast, for the *SUE-Introduce-Present-Respond* (SUE-IPR) *condition* the interviewer introduced the interview in a non-guilt-presumptive way (Phase 1), presented the statement-evidence in/consistencies in a manner that allowed the suspects to comment on these, and then responded to their comments (Phases 2 and 3); all these steps were implemented in a non-judgmental manner (see detailed descriptions below). Phase 4 of the interview concerned the actions for which the interviewer lacked information. This phase was approached in the same manner for all three interview conditions: The interviewer began with an open-ended invitation, and if the suspects revealed information the interviewer asked follow-up questions. Compared to previous studies (Tekin et al., 2016) the SUE-IPR condition is novel, whereas similar versions of the SUE-C condition and Early disclosure condition were also used before.

As in previous studies using the SUE framework to elicit new information (Tekin et al., 2015, 2016), all mock suspects in this study were guilty. The rationale behind this was that previous studies using a similar design have shown innocent suspects to be forthcoming to a very high extent (Luke et al., 2014; Tekin et al., 2014), and we had no reason to assume that innocent suspects would be less forthcoming in the present study. Furthermore, we mapped only the incriminating information that the suspects revealed during the interview and which was new to the interviewer. We used the term “new information” (instead of “admissions” as used in previous studies; e.g., Tekin et al., 2016) as in some countries admissions refer to suspects' statements in court.

In the present study, we introduced a new way to capture the course of the suspects' perceptions of the interviewer's knowledge. That is, the participants listened to the audio recordings of their interviews once the interview was over, and used a checklist to report their perceptions of the interviewer's knowledge at four different points of the interview (after Phase 1, 2, 3 and 4). This procedure enabled us to examine—in a more detailed manner—how specific interview phases affected the suspects' overestimations of the interviewer's knowledge about the critical phase of the crime.

On the basis of previous research and the arguments outlined above, we predicted that both SUE conditions would result in more statement-evidence inconsistencies compared to the Early disclosure condition (Hypothesis 1). Furthermore, we predicted that the SUE conditions would elicit more new information about the phase for which the interviewer lacked information, compared to the Early disclosure condition (Hypothesis 2a). Moreover, we predicted that the SUE-IPR condition would result in more new information compared to the SUE-C condition (Hypothesis 2b). The rationale for this was that we expected the non-judgmental approach (used for the SUE-IPR condition) to increase the suspects' willingness to explain the inconsistencies to the interviewer in Phase 3. In turn, this forthcomingness during Phase 3 was expected to be associated with the suspects' forthcomingness during the critical Phase 4, and consequently the amount of new information elicited.

Furthermore, we predicted that during interview Phase 3 (Hypothesis 3a) and Phase 4 (Hypothesis 3b) the suspects in the SUE conditions would overestimate the interviewer's knowledge about the critical phase of the crime to a higher degree than the suspects in the Early disclosure condition. Finally, we predicted that the suspects in the SUE-IPR condition would perceive that the interviewer had behaved more respectful (Hypothesis 4a), and friendlier compared to the suspects in the SUE-C condition and the Early disclosure condition (Hypothesis 4b). These expectations were based on the non-judgmental approach used for the SUE-IPR condition.

## Methods

### Participants

The study included 88 participants (50 females and 38 males; 69 students, 13 employees and six unemployed persons). The participants' mean age was 27.91 years (*SD* = 9.45; ranging from 18 to 66), and the participants were randomly assigned to one of the three interview conditions (30 for the SUE-C, 29 for the SUE-IPR, and 29 for the Early disclosure). The participants' age, gender and occupation did not differ across the three interview conditions. Originally, 92 participants were recruited, but four had to be excluded as they misunderstood the instructions of the study (three did not follow through with the mock-crime and one used the first possibility to make a full confession). The participants received compensation in the form of course credit or €10. Ethical approval was not required for this study in accordance with the national and institutional guidelines.

### Procedure

#### The mock crime

Participants received information that the study was about security measures and detection of criminal activities. They were instructed to imagine themselves being a member of a criminal group, which had to perform a mission consisting of three phases (A, B, and C) in order to prepare an attack. First, the participants left the building at the forecourt Habelschwerdter Allee, had a brief dialog with an accomplice, and received a paper from her with a code for a locker (Phase A). Second, after returning to the building they entered the library, opened a locker with the code, and took out a cloth bag. The bag contained a book (which they could use to disguise him or her as a student) and a key for a lockbox that had been stolen by an accomplice (Phase B). Third, they walked further inside the building to Street-29, opened a lockbox with the key, and collected a mobile phone and a small box. They were told that an accomplice had prepared the mobile phone for the attack and had stolen the vials inside the box from a chemical lab. Next, the participants walked via a ramp to a notice board on the first floor from which they took a booklet with the label “do not remove.” The booklet included building plans of the target location. Finally, they left the building at the forecourt Fabeckstraße, followed a path next to the building, and deposited all the materials under a ventilation machine (Phase C). During the full mock crime, the participants had the written instructions and a map of the premises with them.

After returning to the lab, the participants were asked to sketch their route on a map in order to check if they performed and remembered the actions correctly. They then received written instructions that a suspicious bag has been found and break-ins and thefts have been reported, that the police were investigating this case and that they had been invited to an interview as a suspect. Furthermore, they were instructed to imagine that they had consulted with their lawyer, who informed the police prior to the interview that the client was innocent and willing to make a statement. In order to increase their motivation, the participants were informed that they would receive their compensation only if they convinced the interviewer of their innocence, but in fact all participants received the compensation. The participants took 10 to 15 min to prepare for the interview.

#### The interviews

One male and one female interviewer were trained in conducting the pre-scripted interviews. They were blind to the hypotheses, and conducted approximately the same number of interviews in each condition. All interviews were audio-recorded and their mean duration was 6.93 min (*SD* = 1.56); no difference was found between the interview conditions with respect to the duration of the interviews, *F*_(2, 85)_ = 1.68, *p* = 0.19, η_*p*_^2^ = 0.04.

**Figure 1:**
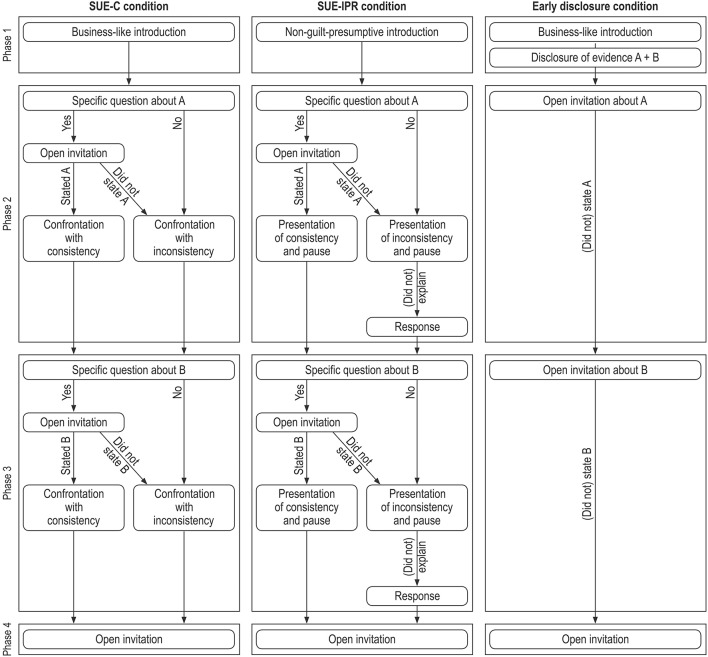
Process of the three interview conditions.

The interviewer held evidence on the crime Phases A and B; that information casted suspicion on the suspect, but did not prove any criminal activity. Specifically, the police had eyewitness evidence regarding Phase A stating that the suspect had (1) been at the forecourt Habelschwerdter Allee, (2) talked to a woman there, and (3) received something from her. The interviewer also had a video from a witness' smartphone about Phase B showing that the suspect had (4) been at the library, (5) opened a locker, and (6) taken out a cloth bag. All interview protocols were divided into four phases: an introductory phase (Phase 1) and three phases of questioning (Phases 2, 3, and 4). See Figure 1 for an illustration of the interview protocols.

#### SUE-confrontation (SUE-C) condition

In this condition, the interviewer started the interview by outlining the suspicion against the suspect, and explained the format of the interview in a business-like manner (Phase 1): “Your lawyer informed us that you are willing to make a statement and that you say you are innocent. I want to ask you a couple of questions about this matter, and it is very important that you answer my questions in as much detail as possible.” The structures of Phases 2 and 3 were identical: The interviewer first asked a specific question about the suspect's whereabouts (e.g., “Have you been outside the building at the forecourt next to the Habelschwerdter Allee?”*)*. If the suspect confirmed being there, the interviewer continued with an open-ended invitation (“Please tell me everything you have done there outside; start with your arrival at the forecourt.”), followed by a follow-up question. Then, depending on the suspect's response, the interviewer disclosed the evidence. Specifically, if the suspect's statement was consistent with the evidence, the interviewer disclosed this statement-evidence consistency in a neutral manner (e.g., “What you say fits well with the statement of a female witness who said that you have talked to a woman outside the building and that she has given something to you. Moving on to my next question now.”) If the suspect's statement was inconsistent with the evidence, the interviewer confronted him or her with this inconsistency by emphasizing the seriousness of this (e.g., “Well, but we have a female witness, who said that you talked to a woman outside the building and that she has given something to you. It is obvious that you are withholding information from me. This is serious and we will return to this later. But now I will move on to my next question.”) Importantly, the interviewer gave the suspect no chance to comment on the in/consistency, instead she continued immediately with posing questions. If the suspect disconfirmed the initially asked specific question about his or her whereabouts, the interviewer directly confronted him or her with the statement-evidence inconsistency. The interviewer used the evidence pertaining to Phase A in Phase 2, and the evidence about crime Phase B in Phase 3. For Phase 4, the interviewer began with an open-ended invitation about the suspect's further actions (“Tell me everything you have done after leaving the philological library and before arriving to the laboratory.”) If the suspect volunteered a clue, the interviewer then invited him or her to explain this in more detail (“You mentioned that you have been on the first floor; tell me everything you have done there.”) Finally, the interviewer asked a follow-up question and closed the interview.

#### SUE-introduce-present-respond (SUE-IPR) condition

The SUE-IPR condition differed from the SUE-C condition with respect to the introduction (Phase 1) and the disclosure of the statement-evidence in/consistencies (Phases 2 and 3). Specifically, the interviewer introduced the interview in a non-guilt-presumptive and non-judgmental manner (Phase 1): “Your lawyer informed us that you are willing to make a statement and that you say you are innocent, and if this is true, you should of course not be here. I am really interested in your point of view in this matter as it is my task to solve this case, and if you are innocent then it is certainly my duty to show this, but then I need your assistance; it is important that you present your account and explanations, and answer and comment on everything as detailed as possible, and that you dispel the existent suspicion and uncertainty, OK?” In Phases 2 and 3, the interviewer presented the in/consistencies in a non-judgmental manner: if the suspect's statement was consistent (e.g., “What you say fits well with the statement of a female witness, who said that you have talked to a woman outside the building and that she has given something to you.”) or inconsistent with the evidence (e.g., “Well, but we have a female witness who said that you have talked to a woman outside the building and she has given something to you.”) Then the interviewer paused for a few seconds to give the suspect the chance to comment without putting pressure on him or her. The interviewer noted the suspect's comment and responded still in a non-judgmental manner if the suspect's explanation was consistent with the evidence (e.g., “OK, now what you say fits well with the witness statement.”) or if it was still inconsistent (e.g., “OK, we might have to look at this more thoroughly then.”). The questioning procedure of Phase 4 was identical to that used for the SUE-C condition.

#### Early disclosure condition

In this condition, the interviewer started the interview exactly as in the SUE-C condition in a business-like manner. However, before posing any questions, the interviewer disclosed all the available evidence (Phase 1): “Ok, we have a female witness, who said that you have been outside the building at the forecourt next to the Habelschwerdter Allee, and she said also that you talked to a woman and she has given something to you. Also, we have a male witness, who said that you were at the philological building and that you opened a locker there and gathered a cloth bag.” For Phase 2, the interviewer began with an open-ended invitation about the evidence pertaining to the suspect's crime Phase A (e.g., “Please tell me everything you have done at the forecourt next to the Habelscherdter Allee. Start with your arrival at the forecourt.”), and a follow-up question. For Phase 3, the interviewer asked questions about the evidence pertaining to the suspect's crime Phase B. The procedure of Phase 4 was identical to those used for the SUE conditions.

#### Post-interview questionnaire

After the interview, the experimenter came into the room, explained that the role-play was over and asked the participants to fill out a questionnaire. The first part of the questionnaire consisted of questions about demographic information (age, sex, and occupation). Then, the participants rated on 7-point scales how motivated they were to carry out their role as a mock criminal during the crime and interview (1 = *Not at all motivated*, 7 = *Very motivated*), and how difficult it was to understand the instructions of the study (1 = *Not at all difficult*, 7 = *Very difficult*). Next, they answered the following two questions about their perceptions of the interview on 7-point scales: “How respectful was the interviewer to you?” (1 = *Not at all respectful*, 7 = *Very respectful*); and “How friendly was the interviewer?” (1 = *Not at all friendly*, 7 = *Very friendly*).

Afterwards the experimenter played to each participant the audio recording of the interview conducted with him or her. The recording was paused four times: (1) after Phase 1, (2) after Phase 2, (3) after Phase 3, and (4) after Phase 4/ the full interview. At each pause, the participants were asked the following question: “When you think back at this point of the interview and consider the interviewer's case-related knowledge, did you think that the interviewer held information that you had not told him or her?” If the participants confirmed this, they were asked to mark, on a checklist with 17 pieces of information, the pieces they perceived the interviewer to know at that specific point of the interview. Six of these 17 pieces were the evidence held by the interviewer (about Phases A and B of the crime). The remaining 11 pieces of information concerned the critical phase of the crime that was actually unknown to the interviewer and which were coded as new information (see below). For the analysis, we used only the participants' perceptions of the interviewer's knowledge about the critical phase of the crime. As the interviewer actually lacked any information of this critical phase of the crime, the suspects could only overestimate the interviewer's knowledge (and not underestimate it).

#### Codings

The suspects' statements were coded concerning the number of *statement-evidence inconsistencies* in Phases 2 and 3. Contradictions and omissions were counted as inconsistencies. The number of inconsistencies with the evidence for both Phases 2 and 3 varied between 0 (no inconsistency) and 3 (inconsistent with all 3 pieces of evidence); thus, the total number of inconsistencies was between 0 and 6. Two persons blind to the experimental hypotheses coded a random 33% of the interviews and on this basis the inter-rater reliability was calculated (Cohen's κ = 0.907). Furthermore, for the SUE-IPR condition, the two persons coded these interviews with respect to the number of inconsistencies that were explained by the suspects (explaining means that the suspect clarified the presented inconsistency); inter-rater reliability was assessed (Cohen's κ = 0.696).

To measure *new information* the interviews were coded with respect to the information revealed for the critical phase. The actions that each suspect had performed during this particular phase were broken down into a total of 11 pieces of critical information. These 11 pieces of information were: (1) walking through Street-29; (2) standing at the lockboxes; (3) taking something from a lockbox; (4) walking over a ramp; (5) being on the first floor; (6) standing at a bulletin board; (7) taking something from the bulletin board; (8) walking over the forecourt Fabeckstraße; (9) walking on a path next to the building; (10) standing next to a ventilation machine; and (11) depositing something under the ventilation machine. Hence, the total number of new pieces of information that a suspect could reveal could vary between 0 (no new information revealed) and 11 (all new information revealed). The same two coders who were blind to the experimental hypotheses rated the 33% of the interviews with respect to the new information revealed and inter-rater reliability was calculated (Cohen's κ = 0.879). All disagreements were discussed, and then one of them coded the remaining interviews.

## Results

### Preliminary analyses

The participants were highly motivated to perform their role as mock criminals (*M* = 5.92, *SD* = 1.05); no difference was found between the three interview conditions, *F*_(2, 85)_ = 1.63, *p* = 0.202, η_*p*_^2^ = 0.04. Furthermore, the participants reported that it was rather easy to understand the experimental instructions (*M* = 2.28, *SD* = 1.24); no difference was found between the three interview conditions, *F*_(2, 85)_ = 0.87, *p* = 0.421, η_*p*_^2^ = 0.02.

### Statement-evidence inconsistencies

A mixed-design analysis of variance (ANOVA) with Interview condition as the between-subjects factor and Phase (2 and 3) as the within-subjects factor was conducted (see Table 1 for the descriptive statistics of the suspects' verbal responses). We found a main effect of Interview condition, *F*_(2, 85)_ = 6.10, *p* = 0.003, η_*p*_^2^ = 0.13. In line with Hypothesis 1, Bonferroni tests showed that the SUE-C condition, *p* = 0.009, and the SUE-IPR condition, *p* = 0.011, resulted in more inconsistencies compared to the Early disclosure condition. No difference was found between the two SUE conditions. There was a significant main effect of Phase, *F*_(1, 85)_ = 15.12, *p* < 0.001, η_*p*_^2^ = 0.15, indicating that Phase 2 (*M* = 1.17, *SD* = 0.09) resulted in more inconsistencies compared to Phase 3 (*M* = 0.78, *SD* = 1.00). Examining this further, we found that the SUE-C condition, *F*_(1, 85)_ = 4.64, *p* = 0.034, η_*p*_^2^ = 0.05, and the SUE-IPR condition, *F*_(1, 85)_ = 12.85, *p* = 0.001, η_*p*_^2^ = 0.13, resulted in more inconsistencies in Phase 2 compared to Phase 3. No such difference was found for the Early disclosure condition, *F*_(1, 85)_ = 0.99, *p* = 0.322, η_*p*_^2^ = 0.01. Finally, no interaction effect was found, *F*_(2, 85)_ = 1.69, *p* = 0.192, η_*p*_^2^ = 0.04.

**Table 1:** Descriptive statistics for the suspects' verbal responses.

**Condition**	**Statement-evidence inconsistencies**			**New information**
	**Phase 2 *M (SD)***	**Phase 3 *M (SD)***	**Phase 2 + 3 *M (SD)***	**Phase 4 *M (SD)***
SUE-C	1.37 (0.93)	1.00 (1.05)	2.37 (1.59)	4.20 (2.54)
SUE-IPR	1.48 (0.91)	0.86 (0.83)	2.34 (1.47)	5.59 (2.97)
Early disclosure	0.66 (0.81)	0.48 (0.83)	1.13 (1.55)	3.79 (2.48)
Total	1.17 (0.95)	0.78 (0.93)	1.95 (1.63)	4.52 (2.75)

### New information elicited

A one-way ANOVA with Interview condition as the factor showed a significant effect on the amount of new information elicited during Phase 4, *F*_(2, 85)_ = 3.60, *p* = 0.032, η_*p*_^2^ = 0.08. Bonferroni tests revealed that the SUE-IPR condition resulted in more new information in comparison to the Early disclosure condition, *p* = 0.037. No significant differences were found between the SUE-C condition and the Early disclosure condition or between the two SUE conditions.

Next, we combined the two SUE conditions (hereafter SUE combined). A one-way ANOVA showed no significant difference between the SUE combined (*M* = 4.88, *SD* = 2.82) and the Early disclosure condition (*M* = 3.79, *SD* = 2.48) with respect to the new information revealed, *F*_(1, 86)_ = 3.12, *p* = 0.081, η_*p*_^2^ = 0.04. Overall, Hypothesis 2a found partial support; Hypothesis 2b found no support.

### Suspects' overestimations of the interviewer's knowledge

**Figure 2:**
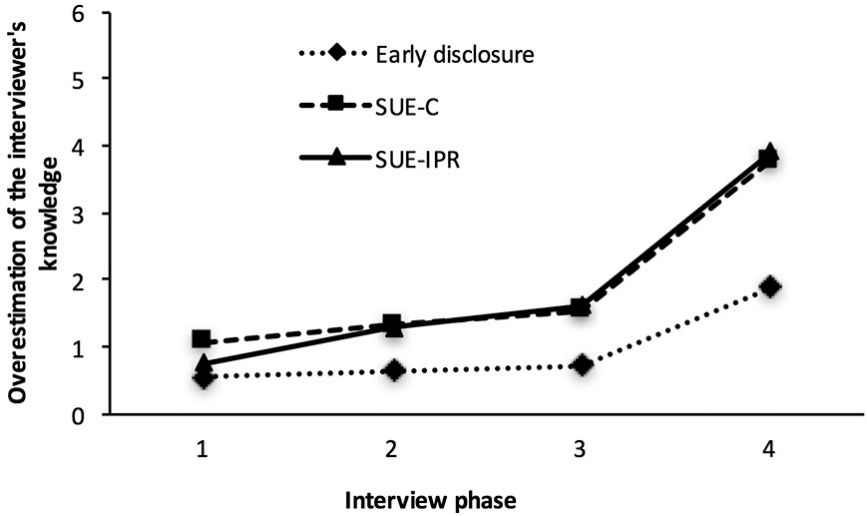
The course of the suspects' overestimations of the interviewer's knowledge about the critical phase of the crime.

We conducted a mixed-design ANOVA with Interview condition as the between-subjects factor and Phase (1, 2, 3, and 4) as the within-subjects factor. The test of sphericity was significant; hence, a Greenhouse-Geisser correction was applied (see Table 2 for the descriptive statistics on the suspects' overestimations). There was no significant interaction effect, *F*_(3.24, 137.84)_ = 2.18, *p* = 0.088, η_*p*_^2^ = 0.05. Furthermore, no main effect of Interview condition was found, *F*_(2, 85)_ = 1.80, *p* = 0.171, η_*p*_^2^ = 0.04. However, there was a significant main effect of Phase on the suspects' overestimations indicating that the extent to which the suspects overestimated the interviewer's knowledge about the critical and unknown phase of the crime changed during the course of the interview, *F*_(1.62, 137.84)_ = 45.54, *p* < 0.001, η_*p*_^2^ = 0.35. Figure 2 illustrates that the suspects' overestimations of the interviewers' knowledge about the critical phase of the crime increased slightly in Phases 1, 2, and 3, and more markedly so in Phase 4. Furthermore, it shows that for the SUE conditions the suspects' overestimations of the interviewer's knowledge about the critical phase of the crime (a) were higher at each interview phase, and (b) increased to a higher extent during the course of the interview, compared to the suspects in the Early disclosure condition.

**Table 2:** Descriptive statistics for the suspects' overestimations of the interviewer's knowledge about the critical phase of the crime.

**Condition**	**Overestimation of the interviewer's knowledge**			
	**Phase 1 *M (SD)***	**Phase 2 *M (SD)***	**Phase 3 *M (SD)***	**Phase 4 *M (SD)***
SUE-C	1.07 (2.83)	1.33 (2.95)	1.53 (3.13)	3.77 (3.65)
SUE-IPR	0.80 (1.50)	1.28 (2.42)	1.62 (2.58)	3.93 (3.45)
Early disclosure	0.55 (1.64)	0.66 (1.65)	0.72 (1.67)	1.90 (2.27)
SUE combined	0.92 (2.27)	1.31 (2.68)	1.58 (2.85)	3.85 (3.47)
Total	0.80 (2.08)	1.09 (2.40)	1.30 (2.54)	3.20 (3.25)

Next, combining the two SUE conditions, we conducted a mixed-design ANOVA with Interview condition (SUE combined vs. Early disclosure condition) as the between-subjects factor and Phase (1, 2, 3, and 4) as the within-subjects factor. Again, the test of sphericity was significant, and we therefore used a Greenhouse-Geisser correction. There was a marginally significant main effect of Interview condition, *F*_(1, 86)_ = 3.64, *p* = 0.060, η_*p*_^2^ = 0.04, indicating that the suspects in the SUE combined (*M* = 1.91, *SD* = 0.29) overestimated the interviewer's knowledge about the critical phase to a higher extent, compared to the suspects in the Early disclosure condition (*M* = 0.96, *SD* = 0.41). Furthermore, there was a significant main effect of Phase, *F*_(1.62, 139.70)_ = 32.42, *p* < 0.001, η_*p*_^2^ = 0.27. Finally, there was a significant interaction effect, *F*_(1.62, 139.70)_ = 4.12, *p* = 0.025, η_*p*_^2^ = 0.05. Simple effect tests showed that for the SUE combined the suspects' overestimations changed during the course of the interview *F*_(3, 83)_ = 20.26, *p* < 0.001, η_*p*_^2^ = 0.42. Specifically, the suspects overestimated the interviewer's knowledge to a higher extent during Phase 3 compared to Phase 1, *p* = 0.013, and during Phase 4 compared to Phase 1, 2, and 3, *p*s < 0.001. For the Early disclosure condition, no such change over the interview was found, *F*_(3, 83)_ = 2.20, *p* = 0.094, η_*p*_^2^ = 0.07. Simple effect tests at each Phase showed that only during Phase 4 the suspects for the SUE combined (*M* = 3.85, *SD* = 3.47) overestimated the interviewer's knowledge to a higher extent than the suspects in the Early disclosure condition (*M* = 1.90, *SD* = 2.27), *F*_(1, 86)_ = 7.54, *p* = 0.007, η_*p*_^2^ = 0.08. Overall, Hypothesis 3a was not supported, and Hypothesis 3b found support.

In general, the degree of the suspects' overestimation of the interviewer's knowledge was positively correlated with the amount of new information elicited (*r* = 0.255, *p* = 0.016). However, no significant correlations were found for the SUE-C condition (*r* = 0.151, *p* = 0.427), the SUE-IPR condition (*r* = 248, *p* = 0.194), the Early disclosure condition (*r* = 0.281, *p* = 0.140), or the SUE combined (*r* = 0.199, *p* = 0.132).

### The suspects' general perceptions of the interview

A one-way ANOVA showed a significant effect of Interview condition on how respectfully the suspects felt that they were treated, *F*_(2, 85)_ = 3.66, *p* = 0.030, η_*p*_^2^ = 0.08. Bonferroni tests revealed that the suspects in the SUE-IPR condition (*M* = 6.38, *SD* = 0.86) felt that they were treated with more respect compared to the suspects in the SUE-C condition (*M* = 5.37, *SD* = 1.88), *p* = 0.025. No difference was found between the Early disclosure condition (*M* = 5.79, *SD* = 1.37) and the SUE conditions. Thus, Hypothesis 4a was partially supported.

A one-way ANOVA revealed a significant effect of Interview condition on the suspects' perceptions regarding the interviewer's friendliness, *F*_(2, 85)_ = 3.50, *p* = 0.034, η_*p*_^2^ = 0.08. The suspects in the SUE-IPR condition (*M* = 5.07, *SD* = 1.41) found the interviewer to be friendlier compared to the suspects in the SUE-C condition (*M* = 4.07, *SD* = 1.62), *p* = 0.030. No differences were found between the Early disclosure condition (*M* = 4.62, *SD* = 1.32) and the SUE conditions. Hence, Hypothesis 4b found partial support.

### Exploratory analysis

Previous studies have shown that suspect who were forthcoming before entering the critical interview phase revealed more new information subsequently (Tekin et al., 2016). Therefore, we examined the effects of the two ways of disclosing inconsistencies (SUE conditions) on the suspects' forthcomingness in Phase 3 more closely. Specifically, we mapped the suspects' forthcomingness in Phase 3 when responding to the interviewer's questions and when explaining the inconsistencies. Furthermore, we examined the influence of the forthcomingness in Phase 3 (i.e., “being in the game”) on the new information revealed in Phase 4.

First, mapping the suspects' forthcomingness in response to the interviewer's questions in Phase 3, forthcoming suspects were defined as suspects who generated no statement-evidence inconsistency when asked questions on the evidence. Conversely, withholding suspects referred to participants who generated a minimum of one statement-evidence inconsistency in Phase 3. A pairwise z-test showed no difference in the proportions of forthcoming suspects between the SUE-C condition (40.0%; *n* = 12) and the SUE-IPR condition (37.93%; *n* = 11), *z* = 0.16, *p* = 0.873. This shows that the elicitation of inconsistencies resulted in a similar number of forthcoming suspects. Further analysis showed that across both SUE conditions, the suspects who were forthcoming at the time when responding to the interviewer's questions in Phase 3 revealed significantly more new information during Phase 4 (*n* = 23; *M* = 6.22, *SD* = 2.88) compared to the suspects who were withholding at that time (*n* = 36; *M* = 4.03, *SD* = 2.47), *t*_(57)_ = 3.12, *p* = 0.003.

Second, we mapped the suspects' forthcomingness in response to the interviewer's questions and when explaining the disclosed inconsistencies (i.e., during the complete Phase 3). Forthcoming suspects were defined as participants who generated no statement-evidence inconsistency or explained at least one inconsistency in Phase 3. In contrast, withholding suspects referred to participants who generated a minimum of one statement-evidence inconsistency without explaining at least one inconsistency in Phase 3. This procedure aimed to examine a possible influence of the two ways of disclosing inconsistencies on the suspects' forthcomingness. A pairwise z-test showed that the proportion of forthcoming suspects was significantly larger for the SUE-IPR condition (75.9%; *n* = 22) compared to the SUE-C condition (40.0%; *n* = 12), *z* = 2.79, *p* = 0.005. This shows that for the SUE-IPR condition the elicitation and disclosure of statement-evidence inconsistencies resulted in more forthcoming suspects compared to the SUE-C condition. Again, across both conditions, the suspects who were forthcoming during the Phase 3 (*n* = 34) revealed significantly more new information during Phase 4 (*M* = 5.94, *SD* = 2.79) compared to the suspects who were withholding during Phase 3 (*n* = 25; *M* = 3.44, *SD* = 2.20), *t*_(57)_ = 3.72, *p* < 0.001. This indicates that it was crucial that the suspects were “in the game” in Phase 3 in order to reveal new information in Phase 4.

## Discussion

This study was on guilty suspects in denial and examined the effects of three modes of evidence disclosure. Overall, we found that when the principles of the SUE framework were used in a non-judgmental manner comparatively more new information was elicited.

### Suspects' verbal behavior

Evidence can be disclosed in different ways. An important factor is the timing of the evidence disclosure. It was found that asking questions about the evidence before disclosing it (SUE conditions) resulted in more statement-evidence inconsistencies compared to when the evidence was disclosed before asking questions about it (Early disclosure condition). This result is in line with previous studies (Tekin et al., 2015, 2016), and can be explained by acknowledging the SUE principles. The suspects in the SUE conditions initially employed withholding counter-interrogation strategies as they perceived the interviewer to be unaware of the evidence that s/he was asking about. In contrast, the suspects in the Early disclosure condition were made aware that the interviewer had knowledge about the evidence, and therefore used more forthcoming counter-interrogation strategies. Also consistent with previous findings (Tekin et al., 2015, 2016), for the SUE conditions the number of inconsistencies declined from Phase 2 to Phase 3. This suggests that after being faced with inconsistencies in Phase 2, the suspects in the SUE conditions might have been revising their perception of the interviewer's knowledge. Based on this revised estimation they might have decided to use a less withholding counter-interrogation strategy for Phase 3.

The elicited statement-evidence inconsistencies can be handled in different ways. This study examines two modes of how to introduce and disclose the in/consistencies. The first was a confrontational way, where the interviewer started in a business-like manner and disclosed the in/consistencies without giving the suspects the opportunity to comment on them (SUE-C condition). The second was a non-accusatorial way, where the interviewer started in a non-guilt-presumptive manner, presented the in/consistencies in such a way that the suspects could comment on them, and then responded to their comments; critically, all steps were used in a non-judgmental manner (SUE-IPR condition). This non-judgmental presentation of the inconsistencies (SUE-IPR condition) resulted in a higher proportion of forthcoming suspects during Phase 3, compared to the confrontational approach (SUE-C condition). This is important as previous findings have shown that suspects need to be willing to discuss the evidence with the interviewer in order for the interviewer to subsequently elicit new information (Tekin et al., 2016). Differently put, it was crucial that the suspects used more forthcoming counter-interrogation strategies when discussing the evidence in Phase 3 before entering Phase 4, in which the interviewer asked about the critical phase of the crime.

The SUE-IPR condition resulted in significantly more new information about the critical phase of the crime compared to the Early disclosure condition. Differently put, for the SUE-IPR condition 48% of the suspects told half or more of all information they held on the critical phase of the crime (i.e., 6 pieces or more), whereas the corresponding figure for the Early disclosure condition was 27%. In contrast to previous studies (Tekin et al., 2015, 2016), the SUE-C condition did not result in more new information compared to the Early disclosure condition. A possible explanation for this is that the SUE-C condition resulted in a lower proportion of forthcoming suspects during Phase 3 compared to the SUE-IPR condition. In support of this reasoning, we found that across the two SUE conditions the suspects who were forthcoming during Phase 3 revealed more new information in Phase 4, compared to the suspects who had been withholding in Phase 3. This indicates that the non-judgmental interviewing style in the SUE-IPR condition promoted (a) the suspects' forthcomingness to discuss the evidence with the interviewer in Phase 3, and (b) the amount of new information elicited in Phase 4.

### Suspects' perceptions of the interviewer

In previous studies the suspects' overestimations of the interviewer's knowledge about the critical and unknown phase of the crime were captured by Likert scale ratings (Tekin et al., 2015) or by completing a checklist (Tekin et al., 2016). These studies found that the suspects in the SUE conditions overestimated the interviewer's knowledge about the critical phase of the crime to a higher degree than the suspects in the Early disclosure condition. For the present study the suspects' perceptions of the interviewer's knowledge about the critical phase of the crime were captured at four points during the interview. This novel way of mapping suspects' perceptions resulted in a more detailed examination and advances our understanding of the effects of the SUE-tactics. Three outcomes are outlined below.

First, an argument for disclosing the evidence early in the interview may be to demonstrate the strength of the evidence and that “it is meaningless to deny any wrongdoing” already at the outset (Leo, 1996). This study found no support for such an argument, as in Phase 1 the suspects' overestimations in the Early disclosure condition were even slightly lower than the suspects' overestimations in the SUE conditions (see Figure 2). Second, in Phase 2 and 3 the suspects' overestimations in the SUE conditions increased slightly compared to the previous phases. This indicates that the disclosure of inconsistencies (SUE conditions) increased the suspects' overestimations to a rather small extent. Third, in Phase 4 the suspects' overestimations in the SUE conditions (a) increased significantly compared to the previous interview phases, and (b) were higher compared to the suspects' overestimations in the Early disclosure condition. An explanation for this is that in the SUE conditions the suspects are believed to have read the interviewer's tactic in Phase 2 and 3 (i.e., asking questions about the evidence before disclosing it). Based on this, the suspects in the SUE conditions might have anticipated the interviewer to use the same tactic also in Phase 4.

When relating the suspects' overestimations of the interviewer's knowledge about the critical phase and the amount of new information elicited, we found a weak positive correlation over all conditions. In contrast to previous results (Tekin et al., 2016), no significant correlations were found within the individual SUE conditions. From this it seems that the suspects' overestimations about the critical phase of the crime were not the sole reason behind the amount of new information elicited. Instead, as outlined above, the suspects' forthcomingness during Phase 3 may also have played a crucial role.

Examining the suspects' general perceptions of the interview, we found that the suspects in the SUE-IPR condition felt that they were treated with more respect, and perceived the interviewer as friendlier compared to the suspects in the SUE-C condition. That is, the non-judgmental SUE protocol resulted in a fostering interview atmosphere. Evans et al. (2014) showed that such a fostering interview atmosphere “that facilitates kindness, cooperation, and respect” (p. 871) resulted in an increasing amount of information elicited. Critically, Alison et al. (2013) also found that a neutral and non-judgmental disclosure of inconsistencies was positively associated with an adaptive interpersonal behavior, which in turn reduced the suspects' resistance and increased the amount of information gathered. This speaks to that the non-judgmental elicitation and presentation of statement-evidence inconsistencies (SUE-IPR condition) resulted in a fostering interview atmosphere that facilitated—for some suspects—a shift of counter-interrogation strategy from less to more forthcoming in Phase 3. The interviewer profited from this shift in Phase 4 by collecting new critical information.

### Limitations and future directions

A non-judgmental interviewing style is particularly important in cases for which the evidence indicates (but does not prove) the suspects' involvement in a crime. Then the interviewer needs to be open-minded. However, a risk of an explicit non-guilt-presumptive approach could be that the interviewer only pretends to be open-minded in order to influence the suspect's decisions; for example, the decision to waive his or her right to silence. Importantly, we clearly distance ourselves from such manipulative use of the presented non-judgmental SUE protocol.

The present study comes with some limitations. First, we limited the sample to guilty suspects in denial and focused only on new incriminating information. For future studies, it may be worthwhile to examine the SUE framework (and especially the non-judgmental implementation of it) by interviewing innocent and guilty suspects mapping incriminating as well as exonerating new information. Second, the study is based on a sample that might not be representative of the usual suspects. We assume that in real-life guilty suspects in denial would be comparatively more motivated to develop and employ counter-interrogation strategies. In line with this, field studies have shown that real-life suspects devise and use verbal counter-interrogation strategies (e.g., “Providing well known information”; Alison et al., 2014), and do not generally deny everything. As the SUE tactics are tailored to counteract such verbal counter-interrogation strategies, they might be even more effective in real-life settings compared to laboratory settings. Third, the participants were stipulated that they would waive their right to silence and claim to be innocent after talking to their lawyer. According to the German Code of Criminal Procedure this process is practically possible. However, in real-life suspects can decide themselves whether they use their right to silence or not. The participants in this study did not have this chance, as for the examination of the interview protocols it was vital that all participants stated to be innocent. Fourth, in the present study both contradictions and omissions were counted as statement-evidence inconsistencies. For future, it may be worthwhile to examine the effects of contradictions and omissions on the suspects' perceptions and verbal behavior separately. Fifth, we mapped the suspects' perceptions of the interviewer's knowledge by playing back the interview to the participants and asking them questions about their perceptions. In brief, such a method runs the risk that the participants are influenced by their previous verbal responses. We aimed to counteract this by carefully instructing them to answer the questions only based on their perceptions at that time of the interview. Overall, we consider this approach as a feasible way to map the suspects' overestimations of the interviewer's knowledge across an interview.

## Conclusions

In suspect interviews the evidence can be used in different ways. This study showed that the interviewer can use the evidence to “keep the suspect in the game” and as a result of this collect new critical information at a later stage of the interview. However, this requires more than just disclosing evidence. In fact, the interviewer needs to elicit and present statement-evidence inconsistencies in a non-judgmental manner. In turn, this will foster a positive interview atmosphere, and affect the suspects to overestimate the interviewer's knowledge and to use more forthcoming counter-interrogation strategies. The non-judgmental SUE protocol can be described as an information-gathering interviewing style that views the suspect in a non-guilt presumptive manner, and thereby our information-gathering approach fits with more general frameworks for investigative interviewing such as the PEACE model (e.g., Milne and Bull, 1999).

## Ethics statement

Structurally similar studies have been conducted at the Psychology Department for 15 years (40 + individual studies), using—more or less—the same paradigm and experimental setup. The Regional Ethical Review Board in Gothenburg (Sweden) has explicitly and repeatedly informed us that we do not have to run each study (using this paradigm) via them. For those occasions where we submitted an application for a singular study, they have just returned the application with the response “We have received your application, but this study is not necessary to run through the Ethics Board”.

## Author contributions

PAG and LM planned the experiment. LM planned the experimental implementation and ST helped with this. LM collected and analyzed the data. LM wrote the manuscript and all authors (LM, PAG, and ST) revised it in cooperation.

### Conflict of interest statement

The authors declare that the research was conducted in the absence of any commercial or financial relationships that could be construed as a potential conflict of interest.
